# Elderly falls associated with benign paroxysmal positional vertigo

**DOI:** 10.1590/S1808-86942010000100019

**Published:** 2015-10-17

**Authors:** Fernando Freitas Ganança, Juliana Maria Gazzola, Cristina Freitas Ganança, Heloísa Helena Caovilla, Maurício Malavasi Ganança, Oswaldo Laércio Mendonça Cruz

**Affiliations:** 1 MD. ENT. PhD - UNIFESP - EPM, Adjunct Professor of Otology and Neurotology - UNIFESP - EPM. Professor - Graduate Program in Vestibular Rehabilitation and Social Inclusion - UNIBAN; 2 Graduate Student - Graduate Program in Otolaryngology and Head and Neck Surgery - UNIFESP; 3 PhD - Graduate Program in Human Communications Disorders UNIFESP- EPM; 4 Senior Associate Professor Otology and Neurotology - UNIFESP-EPM; 5 Full Professor of Otolaryngology - UNIFESP - EPM. Professor - Graduate Program in Vestibular Rehabilitation and Social Inclusion - UNIBAN; 6 Senior Associate Professor - Head of the Otology and Neurology Program - UNIFESP - EPM

**Keywords:** vertigo, aged, dizziness, accidental falls

## Abstract

Benign Paroxysmal Positional Vertigo (BPPV) can cause falls, especially in the elderly.

**Aim:**

to study whether or not elderly patients with BPPV have a reduction on their falls after the particle repositioning maneuver (PRM).

**Materials and Methods:**

retrospective study including elderly with BPPV who had fall(s) during the last year. All patients were submitted to the PRM according to the affected semicircular canal (SCC). After the abolition of positioning vertigo and nystagmus, the patients were submitted to a 12 month follow-up and were investigated about the number of fall(s). Wilcoxon's test was performed to compare the number of fall(s) before and after 12 months of the PRM.

**Results:**

One hundred and twenty one patients were included in the study. One hundred and one patients presented involvement of the posterior SCC, 16 of the lateral and four of the anterior. We noticed a reduction on the number of falls, with statistically significant difference when all the patients were analyzed together (p<0.001), the posterior canal BPPV patients (p<0,001) and the lateral canal VPPB patients (p=0.002). We also found a tendency of statistically significant difference for the anterior canal BPPV patients (p=0.063).

**Conclusion:**

BPPV elderly patients had indeed a reduction on the number of falls after the PRM.

## INTRODUCTION

Populational aging is happening at an ever growing pace, with a relevant increase in the prevalence of chronic-degenerative diseases. The elderly tend to have multiple comorbidities which worsen major geriatric syndromes such as falls, iatrogenic, dementia, lack of mobility, compromising their autonomy and causing disabilities, frailty, institutionalization and death^1^,^2^.

Falls make up the 6th cause of death in people with more than 65 years of age^3^. It is estimated that 30% of the people above this age range fall at least once a year^4^ and that the falls are responsible for 70% of the accidental death in people with 75 years of age or more^5^.

Age increase is directly proportional to the presence of multiple neurotological symptoms associated with body balance, such as vertigo and other dizziness, hearing loss, tinnitus, changes in body balance, gait disorders and occasional falls, among others^6^. It has been shown that the number of falls is significantly higher in patients with bilateral vestibular dysfunction between 65 and 74 years of age when compared to the elderly in the general population^7^.

The relevance of falls in dizzy elderly has led to the National Campaign to Prevent Falls in the Elderly Population, organized by the Brazilian Society of Otology since 2006^8^. One study about the circumstances and consequences of falls in the elderly with chronic vestibular diseases has reported that the most frequent causal agent was sudden dizziness. Benign Paroxysmal Positional Vertigo (BPPV) represented the most prevalent diagnosis on the populational studied (43.8%)^9^.

BPPV, characterized by brief episodes of vertigo, nausea and/or positional nystagmus upon head movements, is produced by the inadequate presence of statocone particles coming from the utriculus macula floating in the endolymph of the semicircular canal (s) or attached to their cupule^10^.

BPPV is the most common cause of vertigo in adults, and at around 70 years of age, 30% of the individuals had had the disorder at least once^11^,^12^. BPPV is also considered the most common cause of dizziness in the elderly^13^.

Vestibular rehabilitation is known as the preferred treatment option in BPPV^14^. Among the most used procedures are the particle repositioning maneuvers for the vertical and horizontal canals^14^, ^15^, ^16^, ^17^.

A study about falls in elderly patients with BPPV submitted to the statocone repositioning maneuvers could increase the relevance of such treatment in this population, investigating its efficacy not only as to vertigo improvement and extinction of the positional nystagmus, but also in relation to the falls. On the other hand, if a reduction in the number of falls after the BPPV maneuvers is not seen, it could suggest complementary treatment to improve body balance in these patients. In the pertaining scientific literature we did not find any study comparing the number of falls in the elderly with BPPV before and after being submitted to the particle repositioning maneuvers.

The goal of the present investigation is to see whether or not the number of falls in elderly patients with BPPV reduces after particle repositioning maneuvers.

## METHODS

This is a retrospective study in which we analyzed the charts of elderly diagnosed with unilateral BPPV in a single canal, regardless of etiology, recurrent or not, for at least 12 months, diagnosed by the presence of vertigo and positional nystagmus during the Dix, Hallpike maneuver18, regardless of the canal involved and the pathophysiological mechanism, and who had fell the previous year. This study was approved by the Ethics Committee of the university institution where it was held, under protocol number 0325/08.

The patients were analyzed as to the presence of falls in the 12 months prior to the medical consultation and in whom BPPV was diagnosed. All the patients were submitted to treatment by the particles repositioning maneuver, according to the semicircular canal involved and the probably pathophysiological mechanism.

The posterior canal involvement was characterized by vertical upwards and rotational positional nystagmus (counterclockwise in the right side labyrinth and clockwise in the left labyrinth). The anterior canal involvement was characterized by downwards and rotational vertical positional nystagmus (counterclockwise in the right labyrinth and clockwise in the left one). In vertical canal involvement, canalolithiasis was characterized by nystagmus lasting up to one minute and cupulolithiasis, by nystagmus lasting more than one minute^10^,^19^.

The lateral canal involvement was characterized by horizontal positional nystagmus. The most intense downwards geotropic nystagmus with the right ear indicated canalolithiasis on the right side; the most intense downwards geotropic nystagmus with the left ear indicated left lateral canal canalolithiasis; the most intense downwards ageotropic nystagmus with the right ear indicated left lateral canal cupulolithiasis; the most intense downwards ageotropic left year nystagmus indicated right lateral canal cupulolythiasis^10^,^19^.

The patients with vertical canal involvement were treated by means of the Epley maneuver^14^ and those with lateral canal involvement were treated by the Lempert-Wilck maneuver^16^. The maneuvers were done in the same visit that the diagnosis was established and all the patients were instructed to return in one week for symptoms reassessment and repetition of the diagnostic maneuver.

After vertigo and positional nystagmus extinction, the patients were clinically followed up by quarterly returns throughout a 12 month period after nystagmus and positional vertigo extinction and were investigated as to the number of falls during such time.

Those patients with BPPV recurrence were instructed as to return as soon as possible for evaluation and treatment by means of statocone repositioning maneuvers until positional nystagmus and vertigo extinction. Only those cases in which positional nystagmus were seen by the examiner were considered recurrent.

Those patients with contraindications to the statocone repositioning maneuvers, those who did not complete the 12 month clinical follow up, those whom during this period developed some disease or had a worsening of their conditions which could impact body balance such as osteomuscular disorders, orthopedic, rheumatologic, neurological, ophthalmological, diabetes mellitus worsening, alcoholism, psychopathological disorders and other vestibular diseases and those who were submitted to some other means of body balance rehabilitation were taken off the study.

The patients were clinically characterized taking into account the semicircular canal involved (posterior, anterior and/or bilateral) probable pathophysiological mechanism (duct or cupulolithiasis)^19^, age range, gender and number of falls.

We carried out a simple descriptive analysis. The statistical analysis held to compare the number of falls before and after the repositioning maneuvers was carried out through the Wilcoxon non-parametric test for related samples because of the lack of Normal distribution in the Kolmogorov-Smirnov (n>50 cases) and Shapiro-Wilk (n<50 cases) tests. The significance level used for the statistical tests was 5% (a=0.05).

## RESULTS

We assessed 131 patients, 121 were included in the study; eight were taken off because they did not complete the follow up period and two for having developed a neurological disorder during the clinical follow up.

The age of the patients ranged between 65 and 89 years, 71 were women and 50 were men.

Twenty-six patients (21%) had BPPV recurrence in the same canal previously affected, and all improved with a new repositioning maneuver.

One hundred patients had posterior semicircular canal involvement, 16 had the lateral and four the anterior canal.

All the patients had positional nystagmus during the Dix-Hallpike maneuver, matching the canalolithiasis pathophysiological mechanism.

Table 1 shows the number of falls before and after BPPV maneuver, regardless of the semicircular canal affected. We also noticed that there was a statistically significant reduction in the number of falls after the treatment, in the cases with one (p<0.001), two (p=0.006), three (p<0.001), four (p< 0.001) or five (p< 0.001) falls before the repositioning maneuver.

**Table 1:** Number of falls before and after the treatment of Benign Paroxysmal Positional Vertigo in elderly patients by means of the particle repositioning maneuvers, regardless of the semicircular canal involved (n=121).

	BEFORE THE MANEAUVER				AFTER THE MANEAUVER			Wilcoxon Test (p-value)
N of falls	N of patients	Mean (SD)	Median	N of falls	N of patients	Mean(SD)	Median	
1	17	1,00 (0,00)	1	0	13	0,24 (0,44)	0	< 0,001

1	4
2	9	2,00 (0,00)	2	0	5	0,44 (0,53)	0	0,006
				2	1				4
				1	36			
3	43	3,00 (0,00)	3	2	6	1,19 (0,45)	1	< 0,001
				3	1			
				0	8			
4	26	4,00 (0,00)	4	2	17	1,46 (1,07)	2	< 0,001
				4	1			
5	26	5,00 (0,00)	5	1	10	1,62 (0,50)	2	< 0,001
				2	16			

Legend: N: Number, SD = Standard Deviation

Table 2 shows the total number of falls before and after BPPV treatment, considering all the semicircular canals involved, we noticed a significant reduction in the number of falls after BPPV treatment in 65.1% of the cases (p<0.001). There was a significant reduction in the number of falls after BPPV treatment of the posterior semicircular canal in 65.1% of the cases (p<0.001) and BPPV of the lateral semicircular canal in 66.6% of the cases (p=0.002). There was a trend towards a statistically significant difference in the reduction of falls after anterior semicircular canal BPPV treatment in 60.0% of the cases (p=0.063).

**Table 2:** Number of falls before and after the BPPV in the elderly by means of particle repositioning maneuvers, according to the canal affected.

Canal involved	Before the maneuver/ After the maneuver			Number of falls				Wilcoxon test (p-value)
		Total	Mean	Standard deviation	Minimum	Maximum	Median	
Posterior. Lateral and Superior	Before	398	3,29	1,28	1,00	5,00	3,00	< 0,001
	After	139	1,15	0,79	0,00	4,00	1,00	
Posterior	Before	358	3,54	1,11	1,00	5,00	3,00	< 0,001
	After	125	1,24	0,78	0,00	4,00	1,00	
Lateral	Before	30	1,88	1,36	1,00	5,00	1,00	0,002
	After	10	0,63	0,72	0,00	2,00	0,50	
Superior	Before	10	2,50	1,29	1,00	4,00	2,50	0,063
	After	4	1,00	0,82	0,00	2,00	1,00	

Table 3 shows the number of falls in cases of posterior semicircular canal BPPV before and after treatment. There was a statistically significant reduction in the number of falls after treatment in the cases with one (p=0.014), two (p=0.023), three (p< 0.001), four (p< 0.001) or five (p< 0.001) falls before the repositioning maneuvers.

**Table 3:** Number of falls before and after the BPPV in the elderly by means of the Epley maneuver, in the cases of posterior semicircular canal involvement (n=101).

	BEFORE THE MANEAUVER				AFTER THE MANEAUVER			Wilcoxon test (p-value)
N of falls	N of patients	Mean (SD)	Median	N of falls	N of patients	Mean (SD)	Median	
1	6	1,00 (0,00)	1	0	6	0,00 (0,00)	0	0,014
	
2	6	2,00 (0,00)	2	0	4	0,33 (0,52)	0	0,023
				1	2			
				1	34			
3	41	3,00 (0,00)	3	2	6	1,20 (0,46)	1	< 0,001
				3	1			
				0	7			
4	23	4,00 (0,00)	4	2	15	0,48 (1,08)	2	< 0,001
				4	1			
5	25	5,00 (0,00)	5	1	10	1,60 (0,50)	2	< 0,001
				2	15			

Legend: N: Number, DP = Standard deviation

Table 4 shows the number of falls in cases of anterior and lateral semicircular canal BPPV before and after treatment. It was not possible to do a statistical comparison of the number of falls before and after treatment in the cases of anterior and lateral semicircular canal BPPV because of the small number of patients with involvement of these canals.

**Table 4:** Number of falls before and after BPPV treatment in elderly patients by the Lempert-Wilck maneuver in the cases of lateral semicircular canal involvement (n=16) and Epley maneuver, for the anterior semicircular canal (n=4).

Canal affected	Maneuver	BEFORE the maneuver		AFTER the maneuver	
		Number of falls	Number of patients	Number of falls	Number of patients
		1	10	01	6
					4
		2	2	0	1
Lateral	Lempert-Wilck			1	1
		3	1	1	1
		4	2	0	1
				2	1
		5	1	2	1
		1	1	0	1
Anterior	Epley	2	1	1	1
		3	1	1	1
		4	1	2	1

## DISCUSSION

The elderly with BPPV included in the present study were mostly females, similar to what was found in previous studies^20^, ^21^. The association between vestibular diseases and hormonal disorders present in women and the greater concern women have in looking for medical care when compared to men could very well justify such prevalence^22^. In the general population, dizziness is more frequent among women, at a 2:1 ratio^23^ and falls are also more frequent in women than in men^24^, ^25^.

There was a prevalence of posterior canal involvement when compared to the lateral and anterior canals, in agreement with previous studies^21,^^26^, ^27^; the spatial location of the posterior semicircular canal favors the migration of statocone fractions coming from the utriculus^28^.

Canalolithiasis was identified in all the patients, in agreement with Caldas^21^ and Korres, Balatsoura^26^ who reported that this pathophysiological substrate happens in most cases of BPPV.

In one year follow up of our patients, BPPV recurrence after the successful repositioning maneuver was seen in 21.5% of the cases. Recurrence varied between 10.0% and 80.0% of the cases treated according to Brandt et al.^29^ and Simhadri et al.^30^. Semont^31^ reported it in 4.2% of his patients and Baloh^20^ reported a recurrence rate of 50%. Macias et al.^32^ reported 13.5% recurrence of BPPV six months after the repositioning maneuver. Helminski et al.^33^ found 43% BPPV recurrence after the repositioning maneuver, without statistical significance between the patients who did and those who did not do the Brandt-Daroff^34^ exercises daily - as a means to prevent recurrence. BPPV recurrence was estimated in 15.0% of the cases per year according to Nunez et al.^35^, 21.8% as reported by Caldas^21^ and 26.0% by Dorigueto et al.^36^. Such result variability among the authors can be explained by the difference in time and means of patient follow up. We believe that the longer the follow up time of these patients, the greater is the rate of recurrence.

In the present study we observed that 17 (14.0%) elderly patients reported only one fall in the previous year, while most of them: 104 (86.0%) reported two or more falls in the same evaluation period. Gazzola et al.^9^ reported that 53.3% of the patients with chronic vestibular disorders who reported falls in the past 12 months, more than half of those (53.1%) had recurrent falls. It is worth stressing that 31.0% of the elderly in the general population from the metropolitan region of São Paulo reported falls in the previous year, and they recurred in about 35.4% of the individuals^24^.

The falls are strongly associated with a drop in physical skills, which follows the individual's aging process, functionally represented by a reduction or loss of the skills to execute daily functions and demands when facing environmental challenges^24^. The postural control may be influenced by the physiological alterations of aging, chronic disorders, pharmacological interactions or specific dysfunctions. Aging affects all the components of postural-sensorial control (visual, somatosensorial and vestibular), effecter (strength, range of motion, biomechanical alignment, flexibility) and central processing, making the elderly more predisposed to falls because of the extrinsic factors associated to the environment where the individual is, such as, for example, lighting, obstacles, slippery floors and/or slopes, amongst others^6^.

The fact that elderly with BPPV in the present study had a reduction in the number of falls after the treatment carried out by means of particle repositioning maneuvers reinforces the relevant influence of vestibular dysfunctions in the onset and/or worsening of the postural control alterations in these patients, predisposing them to instability and body misalignment, culminating with falls^37^. Corroborating this rationale, Gazzola et al.^9^ reported that among the causes of falls of elderly with vestibular disorders, vertigo was the most common, followed by tripping, slipping, syncope/vision blurring, and reduced attention at the time of the fall, loose knees and a sudden obstacle in the path. Moreover, they also observed that recurrent falls in the elderly with vestibular disorders are those statistically more associated with the onset of vertigo, while a single fall is more associated with slipping - similarly to what happens to the elderly in the general population^38^.

The motor tasks developed by the elderly with vestibular disorders at the time of the fall, for example, walking, going up and down stairs, postural transfer activities and taking a shower^9^, frequently involved are rotation and/or hyperextension of the head, which can cause vertigo and/ or positional nystagmus in patients with BPPV. Cohen^39^ observed that standing up from a chair was difficult for 94.0% of the patients with vestibular disorders and ages between 35 and 82 years, walk in a flat or irregular surface for 81.0% and taking a shower and going up stairs for 75.0% of them. These tasks are made up of activities which require a good working of the systems which participate in body balance, and in the presence of dysfunctions in these systems, such activities may become a challenge and may compromise the postural system and lead to falls^40^.

Falls in the elderly in general may have severe consequences to one's body because of mechanical trauma and the stemming clinical complications with a risk of severe lesions in different organs and death. There is a significant association between the number of falls and restriction of activities after the last fall^41^; Elderly patients with vestibular disorders who suffer two or more falls have a greater restriction on their activities when compared to the elderly with vestibular disorders who suffered one fall^9^. The restricted activities, at least temporarily, may stem from trauma, fear of falling, medical advice and/or coexisting conditions^41^,^42^. For the elderly with chronic vestibular dysfunction, the tasks may become more difficult to be performed, since the environment requires greater postural control^43^, ^44^. Out of the home, the visual field stabilization, head and trunk movement and, above all, dynamic balance to face any obstacle which eventually many appear, are potentially more demanding^39^.

The reduction in the number of falls throughout the 12 months subsequent to the particle repositioning maneuvers found in the present study is corroborated by the improvement in quality of life, vestibular manifestations^45^,^46^ and the postural control performance obtained from posturography^47^, ^48^, ^49^, ^50^, ^51^, which were also seen after this treatment in elderly patients with BPPV.

Despite the clinical improvement obtained with the extinction of the vertigo and positional nystagmus and the relevant reduction in the number of falls of elderly patients with BPPV in the current study after the particle repositioning maneuver of statocones, most of them - 103 (85.1%) continued to fall in the 12 months subsequent to the maneuvers. This is a very important data, since failure of other systems associated with postural control are happening to these patients and must be investigated and treated. Thus, the elderly with BPPV who fell must be assessed as to their body balance in a broader sense, including, for instance, balance functional tests, posturo-graphy, sensorial component evaluation of postural control such as visual acuity, kinetic-postural proprioceptive sensitivity, protective-skin sensitivity, vibratory sensitivity and sensorial interaction tests and effective components - such as muscle strength, muscle and connective tissue flexibility and range of motion.

The treatment of falls in the elderly encompasses aspects associated with prevention, disease control and clinical conditions which can cause falls and, also, extrinsic factors such as environmental risks, besides the specific treatment of alterations found on body balance in each patient^52^. Perracini^53^ mentions numerous recommended interventions to treat risk factors of falls in the elderly in the general population, such as:

1) program to strengthen the quadriceps and ankle dorsiflexors;
2) balance training in relation to the integration of sensorial information, control of stability limit, trunk rotation control and efficacy of motor strategies;
3) fitting and/or prescription of gait support device;
4) fitting corrective lens;
5) prescription and proper use of hearing aids;
6) proper use of psychotropic medication;
7) environmental changes and necessary adaptations by an occupational therapist; and
8) specific pharmacological and physiotherapeutic management of chronic-degenerative disorders. Chang et al.^48^ reported that additional training exercises which stimulate the vestibular system can improve postural control and the gait performance in BPPV patients, who have already been submitted to particles repositioning maneuvers.

The identification of risk factors associated with falls which can be modified by means of specific interventions is essential in the prevention of future episodes as well as managing the rehabilitation process.

Therefore, we stress the need to carry out new studies which can contribute to a more encompassing treatment approach in relation to falls in elderly patients with BPPV.

## CONCLUSION

In elderly patients with BPPV, the number of falls reduces after the particles repositioning maneuvers.
